# Clinical and parasitological features of *Leishmania* infection among gold miners in the Oiapoque basin, an international Brazil-French Guiana border

**DOI:** 10.1371/journal.pntd.0012210

**Published:** 2024-06-26

**Authors:** Pamela Mosquera Atehortua, Amanda Figueira da Silva, Lohaine Mafra, Samyra Almeida-da-Silveira, Cintia Xavier De Mello, Hermano Gomes Albuquerque, Lucas André Boaventura de Carvalho, Louise Hureau-Mutricy, Maylis Douine, Alda Maria Da-Cruz, Martha C. Suárez-Mutis, Adriano Gomes-Silva

**Affiliations:** 1 Laboratório Interdisciplinar de Pesquisas Médicas, Instituto Oswaldo Cruz–FIOCRUZ, Rio de Janeiro, Brazil; 2 Laboratório de Doenças Parasitárias, Instituto Oswaldo Cruz–FIOCRUZ–Rio de Janeiro, Brazil; 3 Laboratório de Bacteriologia e Bioensaios, Instituto Nacional de Infectologia Evandro Chagas–FIOCRUZ, Rio de Janeiro, Brazil; 4 Centre d’Investigation Clinique Antilles‑Guyane, Inserm 1424, Centre, Hospitalier de Cayenne Andrée Rosemon, Cayenne, French Guiana; 5 Laboratório de Pesquisa Clínica em Micobacterioses, Instituto Nacional de Infectologia Evandro Chagas–FIOCRUZ, Rio de Janeiro, Brazil; University of Sao Paulo: Universidade de Sao Paulo, BRAZIL

## Abstract

Gold miners working illegally in mines live in poor health conditions related to their strenuous work and precarious housing. Therefore, they are at higher risk for infectious diseases. American tegumentary leishmaniasis (ATL) appears to be of great concern to the population living in the Guiana Shield region. Our aim was to describe their demographic characteristics, the clinical features of cutaneous leishmaniasis (CL), and the frequency of *Leishmania* infection in people working in illegal gold mines in French Guiana. A cross-sectional study was carried out from October to December 2019 in Oiapoque city, Amapá, Brazil. Indeed, many gold miners working in French Guiana are originally from Brazil, and from Oiapoque in particular. A total of 105 participants from 31 different mining sites in French Guiana were recruited. Suspected *Leishmania* infection was confirmed by the following: detection of kDNA in blood or the lesion site; detection of specific antibodies; or detection of IFN-γ release after blood incubation with leishmanial antigens (IGRA-Leish). Nine active CL cases, 38 healed ATL (hATL) and 58 cases with no history of ATL (noATL), were identified. Only half of the treated hATL (50.0%; n = 14) reported having been assisted by a health care unit and the others treated themselves. PCR-kDNA for *Leishmania* was positive in the blood of 100% of CL cases. Curiously, blood PCR-kDNA was positive in 13% of hATL patients and in 15.5% of noATL patients. The IGRA-Leish was positive in 60.5% of hATL and in 37.9% of noATL. In addition to scars suggestive of CL, 71% of hATL had laboratory evidence of *Leishmania* infection. Restriction fragment polymorphism (RFLP) of the *hsp*70 gene identified a sympatric circulation of *L*. *(V*.*) guyanensis* (n = 4), *L*. *(V*.*) braziliensis* (n = 1), *L*. *(L*.*) amazonensis* (n = 2), *L*. *(V*.*) shawi* (n = 1) and *L*. *(V*.*) naiffi/shawi* (n = 1). Taking the laboratory techniques and the clinical evaluations together, 76% (n = 80) of the 105 participants had evidence of *Leishmania* infection. These results suggests that illegal gold miners working in French Guiana are at high risk for infection with different species of *Leishmania*, but their illegal condition and remoteness make it difficult for them to access health services.

## Author summary

American tegumentary leishmaniasis (ATL), a tropical disease, appears to be of great concern to the gold miners working in the Guiana Shield region. Indeed, many gold miners working in French Guiana are originally from Brazil. The frequency of *Leishmania* infection, the clinical features of lesions and scars of ATL, and the demographic characteristics were described among this population. A cross-sectional study was carried out from October to December 2019 in Oiapoque city, Amapá, Brazil. A total of 105 participants were recruited. Suspected *Leishmania* infection was confirmed by the detection of *Leishmania* kDNA or detection of specific immune response to leishmanial antigens. Nine active ATL, 38 healed ATL (hATL) and 58 cases with no history of ATL (noATL), were identified. Only half of the treated hATL (50.0%; n = 14) reported having been assisted by a health care unit and the others treated themselves. Taking the laboratory techniques and the clinical evaluations together, 76% (n = 80) of participants had evidence of *Leishmania* infection. These results suggests that illegal gold miners working in French Guiana are at high risk for infection with different species of *Leishmania*, but their illegal condition and remoteness make it difficult for them to access health services.

## Introduction

Leishmaniasis is a worldwide vector-borne infectious disease associated with poverty. *Leishmania* infection is primarily a zoonosis, and its transmission cycle occurs among wild animals and sandflies. Environmental changes due to anthropic interference interpose humans in the *Leishmania* sylvatic cycle. American tegumentary leishmaniasis (ATL) is endemic in Central and South America, where 1,067,759 cases were reported between 2001 and 2012 [1]. The infection causes a large spectrum of clinical presentations ranging from asymptomatic to cutaneous (localized, disseminated or diffuse leishmaniasis) or mucosal leishmaniasis (ML). Cutaneous leishmaniasis (CL) is the most common form of ATL, which presents quite variable clinical aspects, indicating that in addition to the genetic host background, *Leishmania* strain antigenic differences also drive infection fates. CL can either heal spontaneously or after successful therapy, or evolve to ML. The susceptibility to antileishmanial preconized drugs varies depending on the *Leishmania* species and the geographic genotypic characteristics [2–6]. HIV-coinfected ATL immunosuppressed patients can experience atypical lesions, therapeutic failure or relapses [7–9]. To date, 16 *Leishmania* species belonging mainly to the subgenus *L*. (*Leishmania*) and subgenus *L*. *(Viannia)* have been associated with ATL [10]. Sympatric circulation of *Leishmania* species has been identified in countries covered by the Amazon Basin [11–13].

In the Amazon region, an increase in ATL cases is associated with the expansion of agricultural areas, the occupancy of new areas on the outskirts of cities, the construction of roads, and the establishment of mineral extraction areas such as oil prospecting and gold mining [11,14]. In French Guiana, few mining sites are legalized and have adequate supervision and health policies, but despite this, approximately ten thousand clandestine workers, mainly from Brazil, work in hundreds of illegal mines. Their insecure way of life with poor health conditions related to their strenuous work and precarious housing places them at risk of acquiring tropical diseases, especially ATL, which is strongly associated with environmental degradation in the Amazon rainforest [15–17]. Their remoteness from the health care system hampers access to adequate diagnosis and treatment [18,19].

Although malaria has a high prevalence in gold miners working in French Guiana [20], leishmaniasis is noted as one of the biggest concerns by residents and people who transit through the Oiapoque basin (personal observation). The prevalence of leishmaniasis was 8.3% in gold mine supply sites at the border between French Guiana and Suriname in 2015 [17]. An epidemiological survey of ATL based on records reported by public health services in the Oiapoque municipality (Amapá State/Brazil) from 2008 to 2017 recorded 1,299 new cases, 560 of which were identified as autochthonous for the municipality [15]. The probable infection sites were in the upper Oiapoque basin, and a concentration of ATL cases was found in the illegal gold mines in French Guiana territory. Gold miners comprised 49.14% of cases, which is in line with the high exposure of these subjects related to their labor activity. Particularly, along the Brazil-French Guiana border, *L*. (*V*.) *guyanensis* is the most frequent species, but *L*. (*V*.) *braziliensis*, *L*. (*V*.) *lainsoni*, *L*. (*V*.) *naiffi*, and *L*. (*L*.) *amazonensis* are also found infecting humans [19,21]. However, Brazilian immigration is likely interfering with the dynamics of the *Leishmania* species prevalence over time [21].

In this report, we aimed to evaluate the burden of *Leishmania* infection among Brazilian gold miners who work in French Guiana illegal mines and describe the clinical characteristics of their lesions and the *Leishmania* species circulating in this site.

## Methods

### Ethics statement

This study was conducted in accordance with the Declaration of Helsinki and was approved by the Ethics Committee Board “Comitê de Ética em Pesquisa do Instituto Oswaldo Cruz (CEP FIOCRUZ/IOC)” (number 3.678.242). The subjects were included only after appropriate written informed consent was obtained. The use of *Leishmania* sp in this study was according with the Brazilian Law of Biodiversity and was registered at SisGen (number A022BF7).

### Study design, recruitment strategy and inclusion criteria

The frequency of *Leishmania* infection was investigated in gold mine workers from different mining sites in the French Guiana territory.

A descriptive cross-sectional study was carried out from October to December 2019 in the city of Oiapoque, Amapá, Brazil. The snowball recruitment strategy [22] was employed to invite the subjects. A total of 105 subjects temporarily staying in inns in Oiapoque city were eligible to participate. The inclusion criteria were age over 16 years, presenting any activity related to gold mining in French Guiana, being out of the forest for less than 7 days and an agreement to donate a blood sample. A questionnaire with demographic, epidemiological and clinical data related to *Leishmania* spp. infection risk was completed by the doctor responsible for the clinical examination.

The clinical evaluation included 1) anamnesis with a focus on a history of leishmaniasis or previous antileishmanial treatment; 2) dermatological exams for the detection of scars and cutaneous or mucosal lesions; and 3) images taken of any visible dermatologic lesions.

*Leishmania* infection was screened by 1) PCR for the detection of *Leishmania* DNA in lesions or whole blood; 2) IgG anti-*Leishmania* antigens by ELISA; and 3) *Leishmania-*induced IFN-γ production by lymphocytes in the blood.

**Fig 1:**
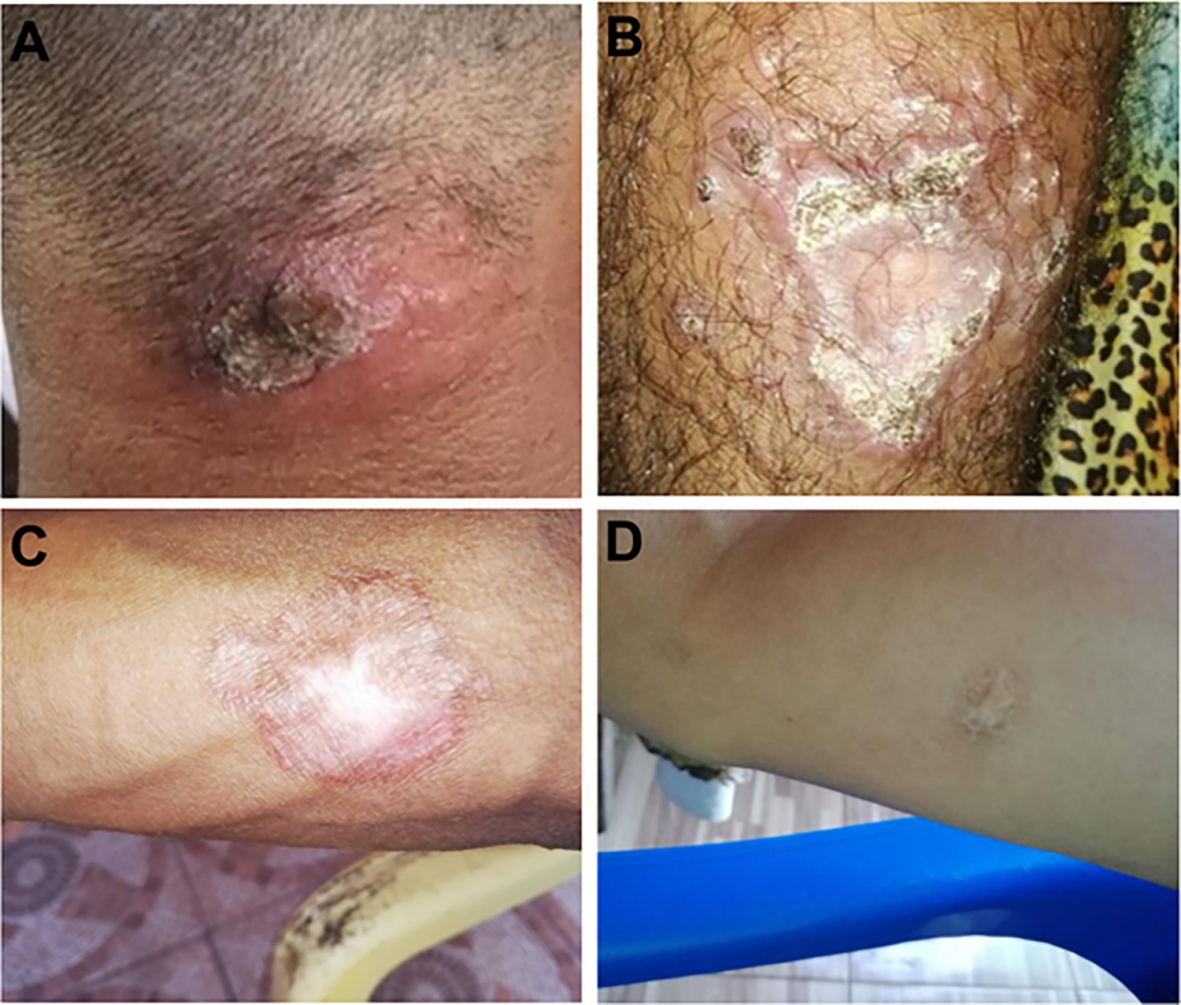
Clinical aspects of cutaneous leishmaniasis lesions in patients from the Oiapoque basin. CL ulcer located on the back of the neck presenting a central crust and perilesional erythema (**A**); CL after treatment with pentamidine located on the left limb–cicatricial central area surrounded by crusty lesions suggestive of reactivation (**B**); Scars with total re-epithelialization of CL located on the right forearm (**C**) and right arm (**D**).

Active CL was defined as individuals with skin ulcers with raised, indurated edges, with a granular base, or reactivating scarred lesions, with crusting and induration, without evidence of mucosal lesions (Fig 1). A healed ATL (hATL) was defined as individuals presenting with skin scars suggestive of CL, with total re-epithelialization of the lesion without desquamation, induration, or any sign of disease activity. Subjects with no previous history of ATL (noATL) were defined as individuals without the presence of active cutaneous or mucosal lesions or scars suggestive of ATL. The asymptomatic subjects were those with noATL who had any positive *Leishmania*-specific immunologic test or kDNA PCR.

### *Leishmania* kDNA detection and *hsp*70 gene species identification by polymerase chain reaction

Two hundred microliters of whole blood and scraping samples from clinical suspicion lesions were employed for DNA extraction using the DNA extraction kit Mini Spin—Kasvi (Kasvi, Mobius Life Science) or the DNeasy Blood & Tissue—Qiagen kit according to the manufacturer’s recommendations.

Initially, a PCR assay targeting the endogenous human β-globin gene [23] was performed to evaluate the extraction efficiency and the presence of inhibitors in the samples. Subsequently, the positive samples in this first assay were subjected to kDNA PCR [24].

Both PCRs were performed in a final volume of 25 μL containing 200 μM dNTPs, 2 U of Taq Polymerase and 1.5 mM MgCl_2_. The cycling parameters were 1 cycle of 94°C/5 min, 35 cycles of 94°C/30 sec, 54°C/1 min, 72°C/1 min, and a final cycle of 72°C/5 min. Positive and negative controls were used in all experiments (100fg of *L.* (*V*.) *braziliensis* (MHOM/BR/75/2903) reference strain were used as positive control, and 2uL of ultra-pure water as negative control). The amplified products were visualized under ultraviolet light on a 2% agarose gel and stained with Gelred (Biotium, Fremont, California).

The positive cases for *Leishmania sp*. were subjected to PCR-RFLP directed toward a fragment of the *hsp*70 gene [25] to identify the *Leishmania* species involved in the infection.

The amplified products were digested with *Hae*III, *Bst*UI and *Mbo*I enzymes according to the manufacturer’s guidelines for the determination of restriction fragment polymorphism (RFLP). Reference strains of *L*. *(V*.*) braziliensis* (MHOM/BR/75/2903), *L*. *(L*.*) amazonensis* (IFLA/BR/67/PH8), *L*. (V.) *guyanensis* (MHOM/BR/1975/M4147), *L*. *(L*.*) infantum* (Syn. *L*. *infantum*) (MHOM/BR/00/1669), *L*. *(V*.*) shawi* (MCEB/BR/1984/M8408), *L*. *(V*.*) lainsoni* (MHOM/BR/1981/M6426), and *L*. *(V*.*) naiffi* (MDAS/BR/1979/M5533) were employed for comparison of the fragment profiles in all PCR-RFLP reactions. The digestion products were subjected to electrophoresis in a 12.5% polyacrylamide gel and silver stained. The sample banding pattern was compared with the profile of the reference strain samples.

### Anti-*Leishmania* IgG, IgG1 and IgG3 detection by ELISA

For antigen preparation, *L*. *(V*.*) guyanensis* (MHOM/BR/1992/IM3862) stationary promastigotes were cultured in Schneider’s insect medium (Sigma Chemical Co., St. Louis, MO, USA) containing 10% fetal calf serum (FCS) (Cultilab, Campinas, BR). After washing with phosphate-buffered saline (PBS), the parasite masses were disrupted with a shaker, and repeated cycles of freezing in liquid nitrogen and thawing at 37°C were performed. The protein content of the total antigen preparation was determined by a Pierce BCA Protein Assay Kit (Invitrogen, Vienna, AT). The measurement of specific antibodies against the *L*. *(V*.*) guyanensis* antigens was performed in accordance with Fagundes-Silva and colleagues (2012) [26]. The cutoff value was determined using the absorbances from positive and negative control serum samples, considering the best AUC in the receiver operating characteristic (ROC) curve analysis (Prism 9.3.1 for Windows, version 9.3.1).

### Anti-*Trypanosoma cruzi* IgG detection by IIF and ELISA

Indirect immunofluorescence was performed in accordance with Camargo 1966 [27], and ELISA recombinant v.3.0 was performed in accordance with the instructions provided by the manufacturers (Weiner lab, Rosario—Argentina). These serological tests were performed to assess the probable cross-reactivity of anti-*T*. *cruzi* antibodies with *Leishmania* antigens.

### IFN-γ release assay specific to *Leishmania* antigens (IGRA-Leish)

For IGRA tests, 1 mL of heparinized whole blood was added to polypropylene tubes containing 50 μg of a disrupted pool of promastigote antigens composed of *L*. *(V*.*) braziliensis* (MHOM/BR/1975/M2903), *L*. *(V*.*) guyanensis* (MHOM/BR/1992/IM3862), and *L*. *(V*.*) naiffi* (MHOM/BR/2010/MS) species (IGRA-Leish*)* or 20 μg of phytohemagglutinin (IGRA-PHA) (Sigma Chemical Co., St. Louis, MO, USA) or an empty tube as a negative control (IGRA-NC). After gentle homogenization by inverting the tubes 10 times, the blood was incubated at room temperature (maximum: 33.0°C; minimum 25.3°C) for 72 hours. The cultures were submitted to additional homogenization procedures every 24 hours. At the end of the incubation, the plasma samples were collected and stored at -20°C for IFN-γ quantification by enzyme-linked immunosorbent assay (DuoSet Human IFN-γ, R&D System, Minneapolis, EUA) according to the manufacturer’s instructions with modifications. The IFN-γ amount was expressed as absorbance values, and the IGRA results were expressed as fold change. The experiment was considered validated when the IGRA-PHA result was higher than 20-fold the IGRA-NC absorbance value. The positive IGRA-Leish results were considered positive when the absorbance values were 5-fold higher than the IGRA-NC absorbance value.

### Data analysis

To conduct comparisons between laboratory results, Fisher´s exact test was used. For the correlation between anti-*Leishmania* reactivity and the time post lesion healing, Spearman’s test was used due to a nonparametric data distribution. All statistical analyses were performed with GraphPad Prism version 9.3.1.

## Results

### Clinical, demographic, and epidemiological data

**Fig 2:**
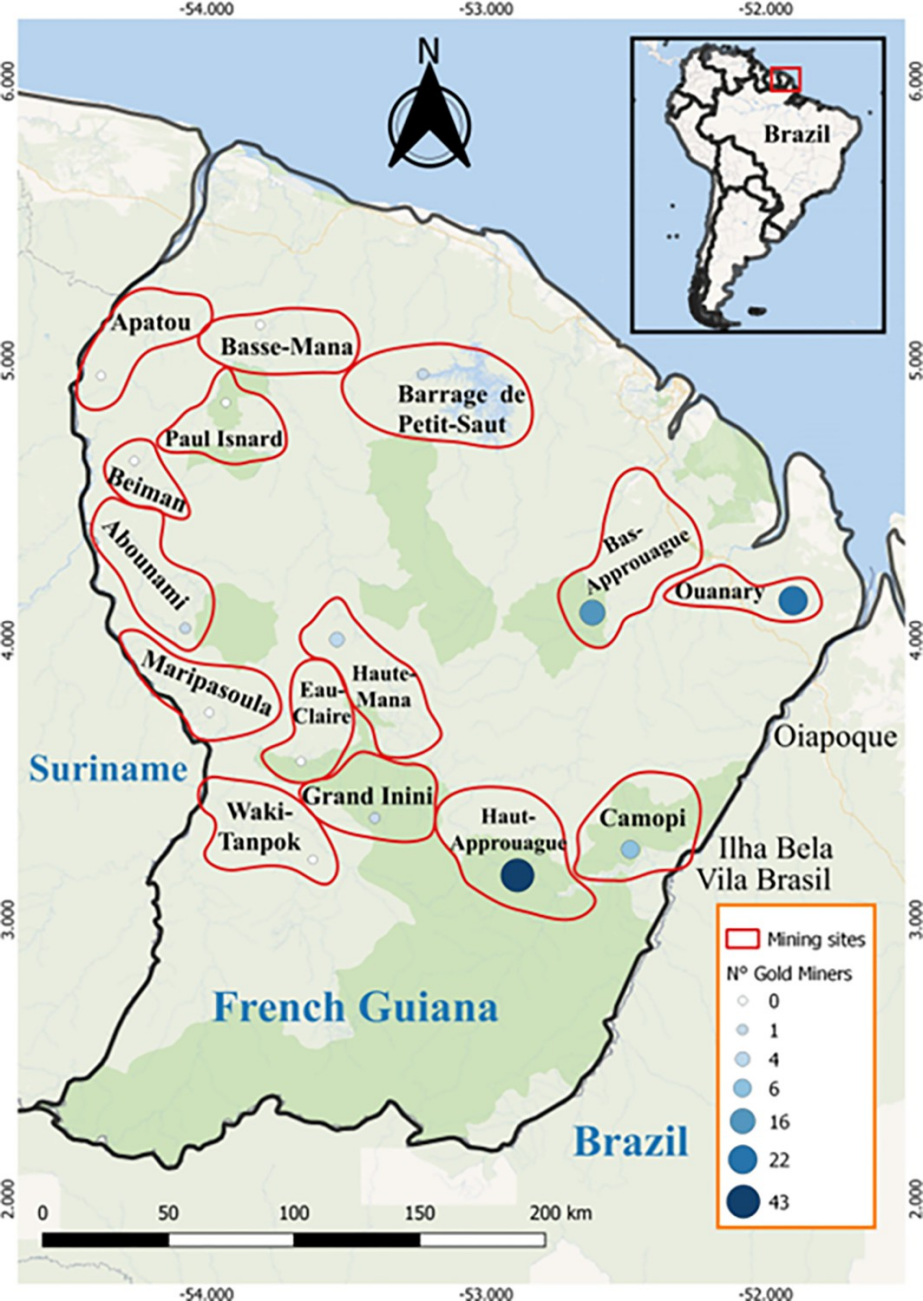
Distribution of gold miners among gold mines regions located in the French Guiana territory. The map was created using open data obtained from "Wikipedia unlabeled layer" from OpenLayers Plugin, Software QGIS 3.28.5. https://maps.wikimedia.org/#9/4.1917/-51.9653.

A total of 105 participants who reported activities related to mining work were recruited. They worked at 15 illegal gold mines in the French Guiana territory (Fig 2), and the most frequent locations geographic regions defined by mining sites referred to by participants were Haut-Approuague (37,1%; n = 43), Ouanary (19%; n = 22), D21 (9%; n = 10) and Bas-Approuague (13,8%; n = 16). Most of the participants directly carried out mining activities within the gold mine (48.5%; n = 51); the others were cooks or janitors (16.2%; n = 17), peddlers (12.4%; n = 13), machine operators (5.7%; n = 6), tankers (3.8%; n = 4), mechanics (1.9%; n = 2), carriers (1.9%; n = 2), loader (0.9%; n = 1), sex worker (0.9%; n = 1), cabaret administrator (0.9%; n = 1), and others for whom we could not find a corresponding English translation (“dala”, “jateiro”, “Maroqueiro”, “piu piu”, “razeiro”, and “valeiro”). The median age was 38 years old (minimum age = 18 years old and maximum age = 66 years old). Most of the study population consisted of men (79.8%). The participants mainly came from Maranhão-Brazil (57.1%; n = 60), followed by Pará-Brazil (20.9%; n = 22). Most of the participants had completed elementary school (57.1%; n = 60), and 10.4% (n = 11) were illiterate. A total of 42.8% (n = 45) reported that they had worked in gold mines for more than 10 years. Approximately 57.1% of the participants perceived ATL as one of the main three health problems in the gold mines in the region.

The participants were subdivided into three groups based on their clinical evidence: active CL (CL, n = 9; 8.6%) (Fig 1); healed ATL (hATL, n = 38; 36.2%), and no previous history of ATL (noATL, n = 58; 55.2%). Among the noATLs, 33 asymptomatic *Leishmania* infections were identified.

All nine CL patients reported gold mining in French Guiana as a possible site of infection. Five presented one lesion, and four had two lesions, which were located mainly on the upper and lower limbs, especially on commonly exposed skin parts. The duration of illness ranged from one month to twelve months (median = 1 month). None of the patients had a previous clinical or laboratory diagnosis of ATL. Five (55.6%) out of nine CL patients reported self-diagnosis and self-treatment with pentamidine purchased on the underground market in the gold mines. All these patients were referred to the local health unit to carry out diagnostic confirmation by direct examination of the lesion and initiate appropriate treatment. Only one participant agreed to receive the health service offered.

The hATL participants presented one to five scars located on the upper limbs (31.6%; n = 12) and on the lower limbs (42.1%; n = 16), and for 10 cases, there were missing data for the place of scars (26.3%; n = 10). Regarding treatment, 40% (n = 16) reported antimonial (Glucantime) as their first drug choice and they were treated with 78 ampoules on average (minimum of 15 and maximum of 200). Two participants did not remember the number of doses. Pentamidine therapy for ATL treatment was reported by 10 participants, with an average use of four ampoules (minimum of one and maximum of 10). Two of these subjects did not remember the drug or how many ampoules they had used. Only half of the treated hATL (50.0%; n = 14) reported having been assisted in a health care unit. Spontaneous CL healing may have occurred in four subjects who reported not having undergone specific treatment for ATL. Three participants reported alternative treatments. No information on treatment was obtained from five participants.

### Laboratory data

*Leishmania* spp. kDNA in venous blood was detected in 21.9% (n = 23) of the total participants and in 100% (n = 9) of the individuals from the active ATL group. This percentage was significantly reduced to 13.1% for the hATL group and 15.5% for the noATL group (Table 1).

**Table 1:** Frequency of positive laboratory results for the detection of *Leishmania* sp. infection in subjects from the Oiapoque basin, Amapá, Brazil.

	Active CL (n = 9)	Healed ATL (n = 38)	noATL history (n = 58)	All participants (n = 105)
Positive kDNA PCR	100% (n = 9) AxH****; AxN****	13,1% (n = 5)	15.5% (n = 9)	21.9% (n = 23)
Positive serology	88.8% (n = 8) AxH**; AxN***	36.8% (n = 14)	24.1% (n = 14)	34.2% (n = 36)
Positive IGRA-Leish	44.4% (n = 4)	60.5% (n = 22) HxN*	37.9% (n = 22)	46.6% (n = 49)

ATL–American tegumentary leishmaniasis; IGRA-Leish—IFN-γ release assay specific to *Leishmania* antigens; n = number

Fisher’s exact test between groups: AxH (active ATL x healed ATL); AxN (active ATL x no ATL history); HxN (healed ATL x no ATL history)

*p<0.05

**p<0.01

***p<0.001

****p<0.0001

**Fig 3:**
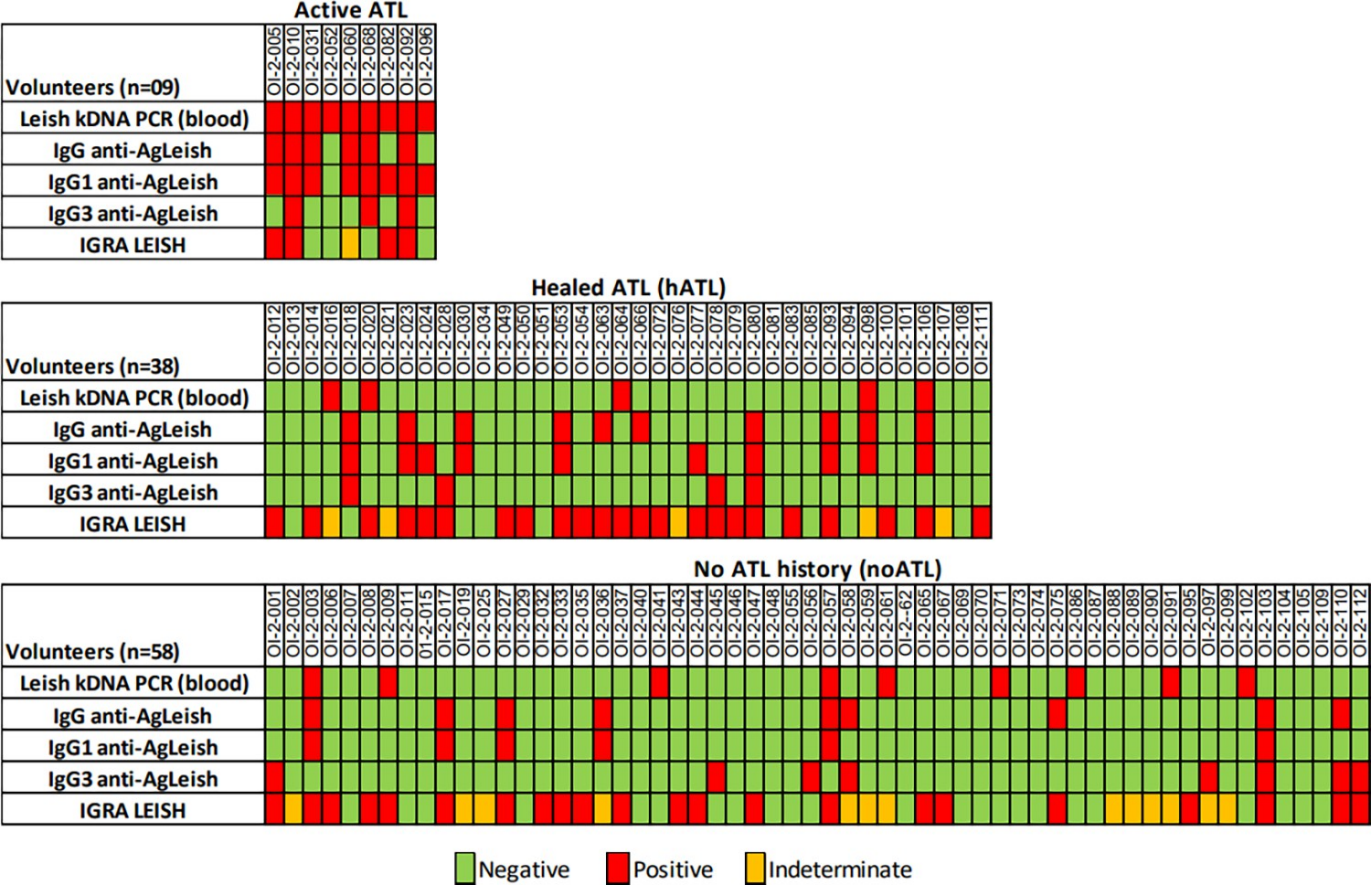
Individual laboratory map to assess *Leishmania* infection. Three assays were employed: detection of *Leishmania* kDNA in blood and cutaneous lesions by qPCR; detection of anti-*Leishmania* IgG, IgG1 and IgG3 by ELISA; and detection of IFN-γ secreted by *Leishmania*-stimulated total blood cells. Red squares represent positive tests; green squares represent negative tests; orange squares represent indeterminate IGRA-Leish results.

At least one positive result for anti-*Leishmania* serology (IgG, IgG1 or IgG3) was observed in 88.8% of participants (n = 8) in the CL group, mainly due to the high positivity for anti-*Leishmania* IgG1 (Fig 3). As expected, the amount of specific immunoglobulin was lower in the hATL group over time post lesion healing, especially for anti-*Leishmania* IgG1, which presented a negative correlation with the time post lesion healing (p = 0.01; r = -0.46; n = 30).

Only one sample showed a positive result on the ELISA test specific to *T*. *cruzi*, concordant with the three specific tests for *Leishmania*, which could be an antibody cross-reactivity with antigens from different protozoa. As *L*. *(V*.*) guyanensis* kDNA was also observed in the peripheral blood of this participant, we cannot rule out the possibility of coinfection with *T*. *cruzi*. Furthermore, the data suggest a low exposure of this population to *T. cruzi* infection.

The specific cellular response to *Leishmania* antigens measured through IFN-γ secretion by blood cells was positive in 46.6% (n = 49) of the participants. The hATL group showed the highest proportion of positive IGRA-Leish tests (60.5%, n = 22), followed by the CL (44.4%, n = 04) and noATL (37.9%, n = 22) groups (Table 1). Different from anti-*Leishmania* serology, the median time post lesion healing was not different between the negative and positive IGRA-Leish subgroups from hATL. Indeterminate results were observed in 18.0% (n = 19) of participants, all of them because the blood IFN-γ concentration in the positive control tube did not reach the minimum established to confirm the viability of the blood cells.

Taking the laboratory techniques and the clinical evaluation together, it was observed that 76% (n = 80) of the 105 participants had evidence of *Leishmania* infection. Among these patients, 58.7% presented CL or scars suggestive of past CL.

**Fig 4:**
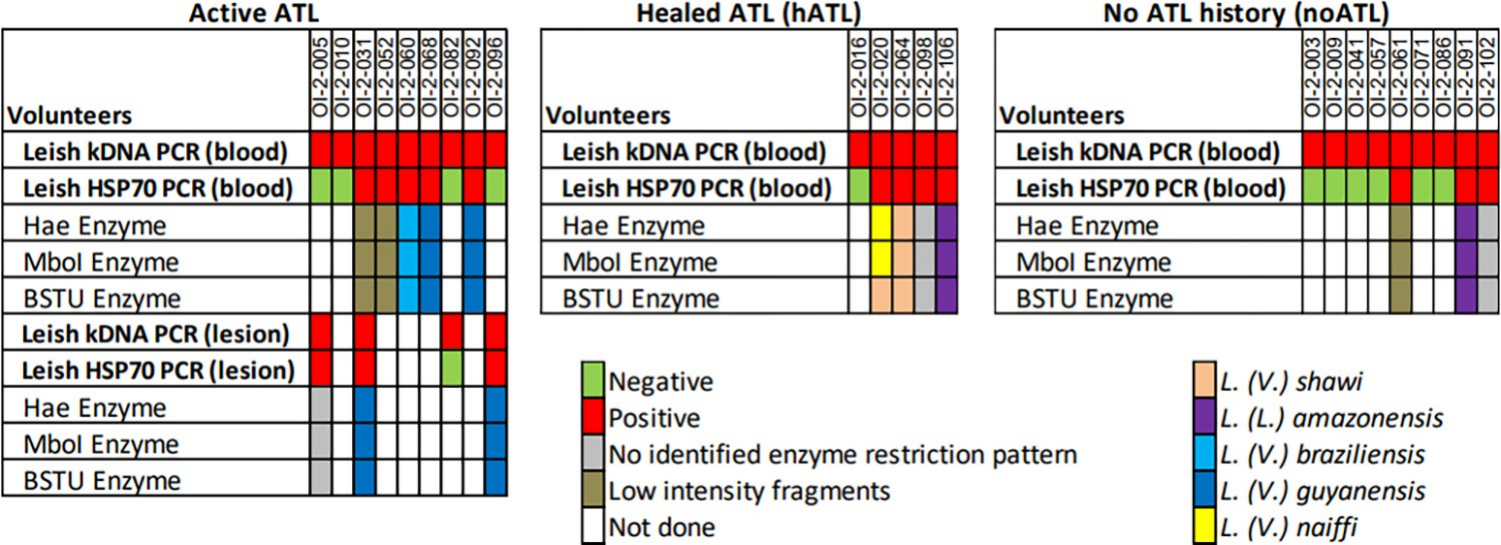
Characterization of *Leishmania* species identified by molecular tests in blood and cutaneous leishmaniasis lesions from individuals with activities related to golden mining in the Oiapoque basin.

Four lesion samples presented 100% kDNA amplification. Among blood and lesion samples with kDNA amplification, 33.0% (n = 9) presented amplification of *hsp*70 (Fig 4). Four *L*. *(V*.*) guyanensis* and one *L*. *(V*.*) braziliensis* were characterized in the CL group. One *L*. *(L*.*) amazonensis* and one *L*. *(V*.*) shawi* were characterized among the hATL group, together with one case of a mixed profile of *L*. *(V*.*) naiffi* and *L*. *(V*.*) shawi*. Finally, one *L*. *(L*.*) amazonensis* was characterized among the noATL. Three samples showed a fragment pattern that could not be identified from the reference strains being used. Additionally, three samples resulted in a low intensity of fragments after enzyme digestion and could not be characterized.

## Discussion

Herein, we associated specific laboratory methods with the clinical data and found that 76% of 105 Brazilian gold miners who worked in French Guiana had evidence of *Leishmania* infection. This proportion was surprisingly elevated, suggesting that people living in gold mines inside the Amazon rainforest are highly exposed to *Leishmania’s* vector. This finding is also consistent with other results [17] showing that more than half of the participants described ATL as one of the main health problems associated with the gold miners’ way of life inside the Amazon rainforest. A possible explanation is that illegal mining activities exert strong pressure on the environment, disturbing adult sandflies after deforestation and allowing massive human-vector contact [16]. It is worth remembering that some of these participants come from endemic regions for ATL and may have been infected before entering the mining regions. Thus, longitudinal studies that can assess local infection need to be conducted to measure this actual exposure to *Leishmania* infection.

The frequency of CL cases observed herein (8.6%) was similar to that found on the Suriname border (8.3%) [17] and was compatible with the number of cases expected during the low transmission season [15], when this study was conducted. The disease characteristics were similar to those observed by Douine and colleagues (2018 and 2022) [17,28], who showed a small number of lesions for most participants. They also observed a high frequency of self-treatments (50% of subjects with an ATL history) with a worrisome number of lesions without complete healing. In the present work, most active CLs (71.4%, n = 5) presented a healing process with relapse signals, which is also characteristic of *L*. *guyanensis* infection [29]. Additionally, the self-treatment for ATL in this population is being performed inadequately at a high economic cost (10 to 15 grams of gold for each ampoule of anti-*Leishmania* agent). Most likely, a subtherapeutic concentration of the drugs or early interruption of treatment had occurred and could have contributed to the scars with relapse signs. The indiscriminate use of anti-*Leishmania* drugs is of great concern due to their systemic toxic effects and the emergence of strains resistant to anti-*Leishmania* drugs [30,31].

The diagnosis of ATL cases is made by clinical evaluation, which can be confirmed by parasitological or molecular tests [2]. However, there is no standard protocol for identifying asymptomatic people for *Leishmania* infection [32]. Many studies have used immunological evidence, such as the Montenegro skin test (MST), to screen these participants, but reagent production has been discontinued due to lack of investment and good production practices [33,34]. Other alternatives, such as serological tests, interferon-gamma release assay (IGRA) or polymerase chain reaction, have been suggested for the identification of asymptomatic ATL infection, but none of them have sensitivity and specificity parameters determined due to the lack of a gold standard test for reference [32]. The IGRA-Leish and MST tests have already been compared to evaluate whether the *in vitro* assay would be able to replace the MST, but the results showed that they are complementary tests in the evaluation of asymptomatic infection by *Leishmania* that causes ATL [35].

Epidemiological surveys to identify people infected with *Leishmania* are commonly performed through serological tests of low sensitivity, especially for recent infections or for individuals with more than two years of successful cure after CL treatment [26,36]. Surveys based on the detection of the cellular immune response have also been reported [37], but due to the discontinuation of the MST antigens, this is no longer an available alternative [34]. Furthermore, the diversity of the immune response induced by *Leishmania* infection, either humoral or cellular, makes the use of a single test capable of indirectly identifying people infected by these protozoa quite challenging, especially in regions where there are multiple species circulating. The association of different techniques based on different principles can increase the ability to identify the infection burden in a population.

Although the present study was carried out at a site of *T*. *cruzi* transmission [38] and there is the possibility of anti-*Leishmania* serology presenting cross-reactivity with antibodies induced by *T*. *cruzi* antigens [39], serological results for *T*. *cruzi* suggest a low interference of cross-reactivity in the results of specific serology for *Leishmania*.

All CL lesions were confirmed by PCR-kDNA in blood. As observed by others [15], we also found a predominance of *L*. *guyanensis* (4 cases), followed by *L*. *braziliensis* (1 case). The PCR results in the blood were surprising for leishmaniasis caused by dermotropic species, and although we did not identify any cases of ML, the hematogenous spread associated with inadequate treatment could contribute to the emergence of the mucosal form of the disease [40–44]. To the best of our knowledge, a few studies have described the use of venous blood from patients with CL for diagnosis based on *Leishmania* kDNA amplification [44,45], but none with high sensitivity was observed in the present work. Additionally, we did not find reports of *Leishmania* kDNA detection in the blood of patients from the Amazon region, where *L*. *(V*.*) guyanensis* is the main species causing disease [46]. *L*. *(V*.*) guyanensis* presents with much evidence of parasite dissemination, as seen by the high frequency of lymphadenopathy [3] and associations with secondary mucosal lesions [7]. Venous blood samples could be a less invasive alternative for obtaining a biological sample to diagnose ATL in this region. But it has limitations such as the low amount of DNA that makes characterization of the species difficult.

The frequency of positive anti-*Leishmania* antibodies was high (in eight of nine patients), especially for IgG1, a Th1-induced IgG subclass. This finding is in accordance with others that showed IgG1 is a promising serologic biomarker for ATL diagnosis [26,36]. On the other hand, the IGRA-Leish results showed that almost half (5 out 9) of the patients had a low capacity to produce IFN-gamma by specific peripheral blood cells, an important effector cytokine induced by *Leishmania* antigens. Three of them were infected by *L*. *guyanensis*, a species that induces low immunological responsiveness characterized by a predominance of the Th2 response [47,48].

IGRA is based on the ability of memory T cells to produce IFN-γ in response to intracellular pathogen-specific antigens and it has been used for the diagnosis of latent *Mycobacterium tuberculosis* infection [49] but is not recommended for active disease. There are few data available on the ability of the IGRA test to identify *Leishmania* infection associated with the visceral clinical form, and no work for ATL was found using total peripheral blood [50]. IGRA evaluation with purified peripheral blood mononuclear cells was suggested as useful for identifying *L*. *braziliensis* infection among subjects with no clinical manifestations of the disease [51]. The healed ATL cases were defined by scars suggestive of CL. Most of them (71.1%) had evidence of *Leishmania* infection confirmed by laboratory tests. The IGRA-Leish was the most effective assay and was positive in 60.5% of the healed ATL participants. This percentage was higher than that observed for active ATL, showing that a clinical cure was accompanied by the maintenance of a parasite-driven Th1 immune response.

A total of 33 out 58 noATLs presented positive laboratory results suggesting infection with *Leishmania* parasites. In our study, these asymptomatic individuals corresponded to 31.4% of the participants, which is the expected ratio for endemic regions of leishmaniasis [52–54]. There is no consensus on the methods that should be used to identify a history of leishmaniasis. The Montenegro intradermal reaction is the most widely used but is currently not available [53]. Thus, our strategy was to combine the clinical data with laboratory methods based on a search for *Leishmania* DNA along with an investigation of specific cellular and humoral immune responses. To the best of our knowledge, this approach has not been employed before, and the use of IGRA-Leish for this purpose can become an important tool to address memory response in leishmaniasis. This technique is easily adapted for field work where a well-equipped laboratory is unavailable.

In the hATL, blood circulation of *Leishmania* DNA could be observed in 13.1% of subjects, which suggests parasite persistence in some individuals without active lesions. Among healed individuals, it is likely that inadequate treatment with medicines purchased on the illegal market and without the supervision of a health professional may contribute to the low efficiency of the anti-*Leishmania* effect. Even after the lesions heal, the persistence of the parasite can put these individuals at risk of future reactivation of the disease or even evolution to the mucosal form [44]. It is worth noting that in a study carried out in Rio de Janeiro, Brazil, an endemic region for *L*. *(V*.*) braziliensis*, enrolling 54 individuals cured for ATL after treatment (low dose or conventional dose of antimonial pentavalent) supervised by health professionals, it was observed that 9.2% of them had detectable *Leishmania* DNA in peripheral blood. Our results based on the participants self-report, showed 5 individuals from hATL who had positive PCR for *Leishmania* kDNA, presented healing time ranging from 18 to 240 months. Among the hATL participants with negative PCR for *Leishmania* kDNA, self-reported time of cure ranged from 9 to 288 months. So, we did not observe any association between parasitological results (PCR) and the healing time. This suggests that regardless of the treatment regimen and time of healing, probable parasite persistence remains in some of these subjects [55].

The limitations of the study include the low number of participants recruited, especially due to the difficulties involved in recruiting the illegal miners working in the border region, so this study population was of convenience and not calculated previously for determine the sample size. The low number of recruitments was also impacted by the exclusion criterion "being out of the forest for less than 7 days", which was adopted because it was a sample originally designed for a study in malaria. The present ATL investigation was later added to the logistics of the malaria study that had already been initiated, with the aim of expanding the investigation of other infectious diseases of importance to the miner population. Thus, we encourage new studies that can more robustly investigate ATL in the population of miners to give us a more accurate diagnosis of the situation, and thus guide health authorities in the implementation of better strategies to control the disease.

The high burden of *Leishmania* infection among the gold miner population from the Brazilian-French Guiana frontier reinforces the necessity of an active health care system that can quickly diagnose suspected cases of ATL, treat them appropriately, and contribute to the control of leishmaniasis in Brazil. Additionally, the necessity of population awareness about the risks of unsupervised treatment of suspected leishmanial lesions should be considered.

We hope that this study can encourage further research to seek answers about ATL in the population of miners that could not be performed yet. Further studies with more detailed clinical manifestations, especially those observed in patients who treated their lesions without appropriate medical supervision, should be conducted. We also hope that this study can stimulate discussions about public policies aimed at this population, and a justification for access to treatment and health more broadly, including alternative therapeutic possibilities for this group of individuals subjected to exceptional working conditions.
